# Primary follicular lymphoma of the epididymis positive for t(14;18)(q32;q21)/*IGH-*BCL2 and negative for BCL2 protein expression: a case report

**DOI:** 10.1186/1752-1947-6-24

**Published:** 2012-01-18

**Authors:** Vincenzo Tralongo, Gaspare Becchina, Claudia Nagar, Gabriella Ottoveggio, Silvia Mason, Barbara Seno, Francesca Sessa, Milo Frattini, Francesco Genovese

**Affiliations:** 1 Department of Diagnostic Laboratory, U.O.C. of Pathologic Anatomy, "G.F. Ingrassia" Hospital, ASP Palermo, Italy; 2 AB ANALITICA s.r.l. Padua, Italy; 3 Laboratory of Molecular Diagnostic, Institute of Pathology, Locarno, Switzerland

## Abstract

**Introduction:**

Non-Hodgkin lymphoma (NHL) can involve the paratesticular organs as the primary disease, as primary testicular lymphoma that secondarily involves the paratesticular structures, as the initial site of presentation of occult nodal disease or as the result of disease dissemination. Primary follicular lymphoma of the epididymis in an adult is extremely rare. Little is known about primary adult paratesticular/epididimal lymphomas.

**Case presentation:**

We report a rare case of primary follicular non-Hodgkin lymphoma of the epididymis in a 90-year-old Caucasian man who presented with a left scrotal mass. Bone marrow biopsy was negative and computed tomography of the total body revealed no evidence of extratesticular involvement. Macroscopically, the epididymis was replaced completely by a uniform mass. Histologic studies revealed a dense lymphoid infiltrate predominantly composed of centrocytes with admixed centroblasts. Immunohistochemical analyses demonstrated that neoplastic cells strongly expressed CD45RB, CD20, CD79a, bcl-6 and CD10; bcl-2 immunostaining was negative. Molecular studies showed the presence of the monoclonal IgH gene rearrangement and the IgH/BCL2 rearrangement. The lymphoma was classified as follicular lymphoma, low grade, grade 1-2. The patient subsequently underwent radical orchiectomy, did not receive chemotherapy and post-operative follow-up showed absence of disease recurrence.

**Conclusions:**

The case of primary follicular lymphoma of epididymis, reported here, is considered a very rare event. It is characterized by clinically indolent localized disease, a good clinical outcome, lack of expression of BCL2 protein and the presence of the t(14;18)(q32;q21)/*IGH-BCL2*. Even if it is a single case, the primary follicular lymphoma epididymis with t(14;18) could represent either a variant of the previously reported t(14;18)-negative primary paratesticular follicular lymphoma or a distinct biological entity. To report additional cases in the future would be helpful in resolving this question.

## Introduction

Non-Hodgkin lymphoma (NHL) can involve the paratesticular organs as the primary disease, as primary testicular lymphoma that secondarily involves the paratesticular structures, as the initial site of presentation of occult nodal disease or as the result of disease dissemination. Primary lymphoma of the paratesticular organs, without testicular or systemic involvement, is an unusual observation [1]. Primary NHL of the epididymis is a rare pathological event and is extremely rare in an adult [2,3]. When present, epididymal involvement is generally associated with testicular primary lymphoma or is a finding of systemic disease [4].

Nodal follicular lymphoma is one of the most common types of NHL and accounts for approximately 20% to 30% of cases in adults, but testicular and paratesticular primary follicular NHL is an unusual event.

Primary epididymal lymphomas often occur in older men, have an intermediate or high grade and follow an aggressive clinical course.

The epidydimis was involved in 16 of 20 cases of testicular lymphoma described by Abel *et al*. [5] and in five of 11 cases reported by Johnson *et al*. [6]; in the series reported by Ferry *et al*. involvement of epididymis was found in 63% of testicular lymphomas. Predominant epididymal involvement may simulate primary epididymal lymphoma: epididymis was more extensively involved than the testis in three of 43 cases reported by Ferry *et al*. [7].

Primary epididymal lymphoma without testicular or systemic involvement was reported by Novella *et al*. [8], Suzuki *et al*. [9] and Inoue *et al*. [10]. In 1979 Schned *et al*., reported a case of primary histiocytic lymphoma of the epididymis [11]. Heaton and Morales reported a second case in 1983 [12] and Ginaldi *et al*. in 1993 described a 68-year-old man with a primary epididymal lymphoma [4]. In 1995 McDermott *et al*. reported a case in a 34-year-old man [13]. Also, in 1995 Kausch *et al*. reported the first case of primary mucosa-associated lymphoid tissue (MALT) of the epididymis [14]. Okabea *et al*. reported a case of primary lymphoma of the spermatic cord with involvement of epidydimis but not testis [15].

To the best of our knowledge, little information about adult primary follicular NHL of the testis and epididymis is available. We report here a rare case of primary follicular NHL of the epididymis in a 90-year-old man with immunophenotypical and genetic characterization.

## Case presentation

A 90-year-old Caucasian man, in otherwise good health, presented with a left scrotal mass that had been present for eight months and was occasionally painful. The man had no fever, weight loss or night sweats; there was no lymphadenopathy, hepatosplenomegaly or mass in the right testis.

The patient underwent left radical orchiectomy. Macroscopically, the specimen consisted of testis, epididymis and spermatic cord. On the cut surface, the epididymis was replaced completely by a uniform tan mass that measured 6 × 3 cm without areas of necrosis or hemorrhage. No invasive lesions were observed in the testis and spermatic cord and the neoplastic tissue was restricted to the epididymis.

**Figure 1:**
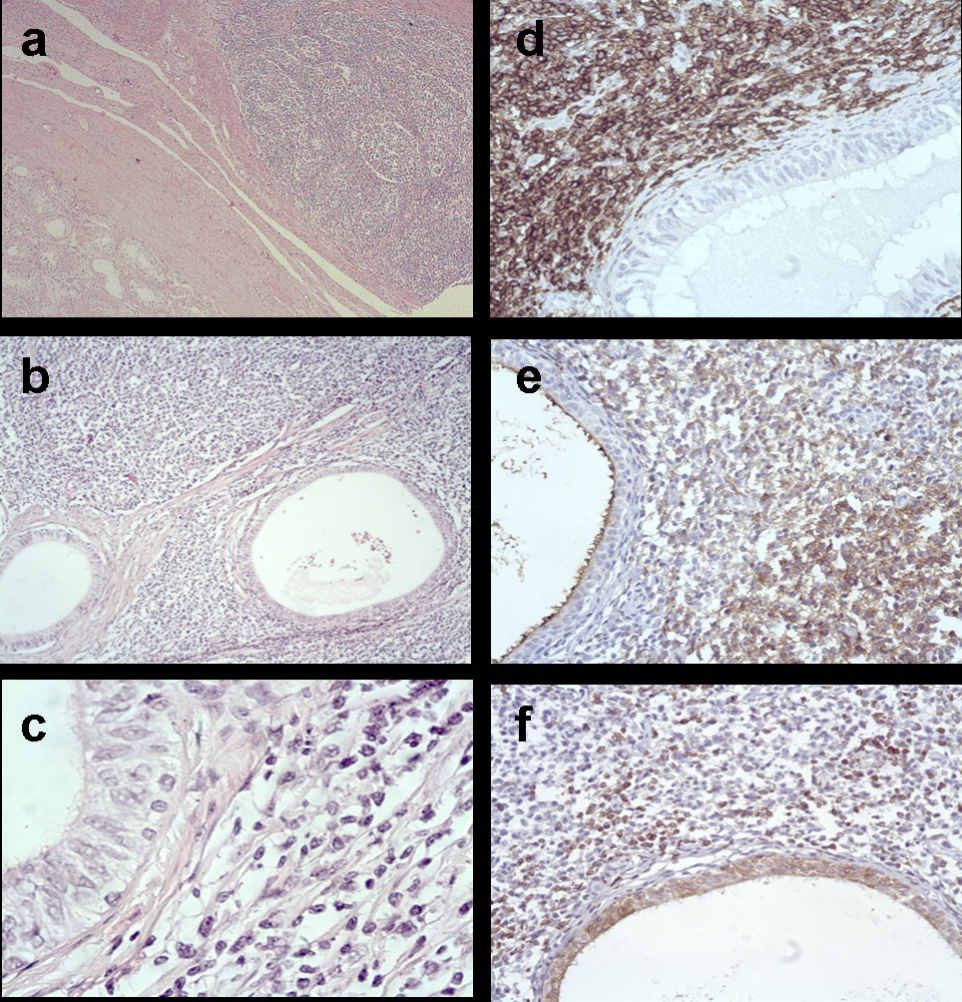
**Histological and immunohistochemical findings**. (**a**) The testicular parenchyma shows no evidence of lymphomatous involvement (hematoxylin-eosin, original magnification ×50). (**b-c**) Residual epididymal tubules are surrounded by a dense cellular infiltrate with a prevalent follicular architecture (hematoxylin-eosin, original magnification ×100 and ×630, respectively). The neoplastic cells strongly express CD20 (**d**) and CD10 (**e**) but are bcl-2 negative (**f**) (original magnification ×250).

Microscopically, the testicular parenchyma showed no evidence of lymphomatous involvement (Figure 1a). The epididymis contained a dense lymphoid infiltrate predominantly composed of small cleaved cells (centrocytes) with admixed small and large non-cleaved cells (centroblasts). The number of centroblasts ranged between five and 15. The neoplasm predominantly displayed a follicular growth pattern (Figures 1b and 1c). Neoplastic follicles were quite expansive and irregular and were coalescent in some areas. Well formed mantle zones were absent. No tangible-body macrophages were present.

**Figure 2:**
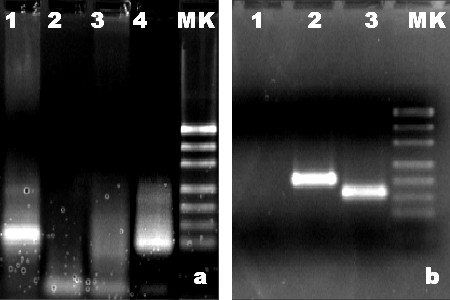
**Molecular findings**. (**a**) Agarose gel electrophoresis to detect the rearrangement of the immunoglobulin heavy chain (IgH) rearrangement PCR products (semi-nested PCR). Lane 1: patient sample; lane 2: negative control (no DNA); lane 3: polyclonal control; lane 4: monoclonal control; lane MK: DNA ladder (501, 404, 331, 242, 190,147,111, 67, 34 bp fragments). (**b**) Agarose gel electrophoresis to detect BCL2/J_H _PCR products (nested PCR). Lane 1: negative control (no DNA); lane 2: patient sample; lane 3: BCL2 MBR positive control (plasmidic DNA containing BCL2/MBR rearrangement); lane MK: DNA ladder (501, 404, 331, 242, 190,147,111, 67, 34 bp fragments).

Immunohistochemistry (IHC) investigation demonstrated that neoplastic cells strongly expressed CD45RB (LCA, clone 2B11+PD7/26, Dako), CD20 (clone L26, Dako), CD79a (clone JCB117, Dako), bcl-6 (clone PG-B6p, Dako) and CD10 (clone 56C6, Dako) (Figures 1d and 1e). On the other hand, staining for CD3 (clone F7.2.38, Dako), CD45RO (clone UCHL1, Dako), CD5 (clone 4C7, Thermo Scientific), CD23 (clone SP23, Dako) and bcl-2 (clone 124, Dako and clone 100/D5, Novocastra) were negative (Figure 1F). Immunostaining for CD21/CD35 (clone 1F8/Ber-MAC-DRC, Dako) demonstrated expanded networks of follicular dendritic cells within the nodules. Approximately 60% of the neoplastic cells reacted positively when stained with antibody against Ki-67 (clone MIB1, Dako). The proliferation index of follicular lymphoma generally correlates with tumor grade. The proliferation index reported here was independently performed by more than one pathologist and, although it is somewhat high for a low grade follicular lymphoma, discordances are known to occur in follicular lymphoma [16]. Finally, polymerase chain reaction (PCR) analysis revealed a monoclonal immunoglobulin heavy chain gene rearrangement and the detection of t(14;18)(IgH/BCL2) revealed the BCL/J_H _rearrangement (Figures 2a and 2b). The lymphoma was classified on the basis of the World Health Organization's (WHO) 2008 guidelines as follicular lymphoma, low grade, grade 1-2, with follicular pattern. The disease was staged as IE (extranodal) primary epididymal lymphoma. Commercial kits by AB Analitica (Padua, Italy) were used to perform both procedures according to the manufacturer's instructions. All used products are compliant with the requirements of the *in vitro *diagnostic directive (IVDD) 98/79/EC. IHC and molecular analyses were carried out as previously described [17-20].

The patient underwent full staging for lymphoma, including computed tomography of the total body which did not reveal any evidence of extratesticular involvement (image not shown) and did not receive chemotherapy. Bone marrow biopsy showed no evidence of lymphoma (data not shown). A post-operative follow-up of more than 15 months has been uneventful, without disease recurrence.

## Discussion

This report describes an unusual case of primary follicular non- Hodgkin lymphoma (NHL) of the epididymis in a 90-year-old man. The diagnosis was supported both by histology and phenotypic or genotypic expression of clonality. Although, the majority of follicular lymphomas arise in the lymph nodes, a minority of cases occurs primarily in extranodal sites, including the gastrointestinal tract, skin, ocular adnexa, breast and testis [21]. Several studies report that primary testicular lymphomas are almost always represented by diffuse large B-cell lymphoma and mainly affect older adults with frequent dissemination and poor prognosis [21-24]. A small number of testicular lymphomas are follicular lymphomas, mainly affecting children [25-29], but adults also develop testicular follicular lymphoma in rare cases [30-34].

After reviewing the literature, we found only a few cases of primary adult paratesticular and/or epididimal lymphomas. These lymphomas have been well characterized.

With regard to clinical behavior, spread beyond the primary site is very unusual for testicular follicular lymphoma and patients with these lymphomas have an excellent clinical outcome [21].

With respect to immunophenotypic and cytogenetic features, some extranodal follicular lymphomas such as those involving the gastrointestinal tract, are similar to those of nodal origin, in which bcl-2 protein is usually expressed and the translocation involving bcl-2 and IgH genes is frequently detected [21].

Other lymphomas, such as those involving the skin and testis, are typically negative for bcl-2 protein and have no bcl-2 gene rearrangement when this has been evaluated [21].

Bacon *et al*. described five cases of primary follicular lymphoma of the testis and epididymis in adults characterized by immunophenotypic expression of CD10 and BCL6, which lacked immunophenotypic expression of BCL2 and lacked t(14;18)(q32;q21)/IGH-BCL2 and BCL6 rearrangements [31]. The same features have been found in lymphomas in children [26-28].

Other studies report that the majority of bcl-2-negative nodal follicular lymphomas lacks a t(14;18) but a significant subset of these tumors are false negative; the presence of somatic mutations in the translocated *BCL2 *gene results in amino acid replacements in the region of the epitope recognized by the antibody [35].

Our case is characterized on histology by the presence of small cleaved cells, displaying predominantly a follicular growth pattern. In addition, we used bcl-2 immunostaining, with alternative monoclonal antibodies (124 and 100/D5 clones), to help in the diagnosis of follicular lymphoma and to reduce false negatives, since reactive follicular center cells are negative for bcl-2. However, extranodal follicular NHL are often non reactive to bcl-2.

In the epididymis, phenotypically we found cells positive for bcl-6 and CD10 and negative for bcl-2.

To confirm the presence of the lymphoma, Ig gene rearrangement analysis using PCR was performed. The analysis showed the presence of the monoclonal IgH gene rearrangement.

Finally, we investigated the t(14;18)(IgH/BCL2) rearrangement, since the bcl-2 gene translocation is present in the majority of nodal follicular lymphomas and is involved in the t(14;18) reciprocal translocation with the IgH locus. Since this translocation was present, we confirmed the diagnosis of follicular lymphoma, low grade, grade 1-2 (WHO 2008).

Lack of expression of the bcl-2 protein simultaneous with the presence of the t(14;18) can be explained by the fact that the traslocation could cause somatic hyper-mutation of the bcl-2 gene sequence encoding the epitope recognized by the antibody, whereby the neoplastic cells appear negative for bcl-2 protein despite having t(14;18).

In conclusion, on the basis of data from our case and from the literature, whatever the roles of protein bcl-2 expression and/or bcl-2 gene rearrangement, there is no significant correspondence with clinical behavior [36].

## Conclusions

The case of primary follicular lymphoma of epididymis, reported here, is considered a very rare event.

It is characterized by clinically indolent localized disease, a good clinical outcome, lack of expression of BCL2 protein and the presence of t(14;18)(q32;q21)/*IGH-BCL2*.

Even if it is a single case, the primary follicular lymphoma epididymis with t(14;18) could represent either a variant of the previously reported t(14;18)-negative primary paratesticular follicular lymphoma or a distinct biological entity (perhaps more closely related to indolent BCL2 rearranged follicular lymphoma in other sites). To report additional cases in the future would be helpful in resolving this question.

## Consent

Written informed consent was obtained from the patient for publication of this case report and any accompanying images. A copy of the written consent is available for review by the Editor-in-Chief of this journal.

## Competing interests

The authors declare that they have no competing interests.

## Authors' contributions

VT analyzed and interpreted the patient data regarding histological and immunohistochemical examination of the epidydimis and wrote the manuscript; CN, GB and GO contributed to the histological examination; SM, BS and FS contributed to the molecular studies; MF contributed to the molecular studies and analyzed data; FG analyzed and interpreted the patient data regarding molecular studies and wrote the manuscript. All authors read and approved the final manuscript. VT and FG contributed equally to this work.
